# An Intimate Relationship Between Eriophyoid Mites and Their Host Plants – A Review

**DOI:** 10.3389/fpls.2018.01786

**Published:** 2018-12-04

**Authors:** Enrico de Lillo, Alberto Pozzebon, Domenico Valenzano, Carlo Duso

**Affiliations:** ^1^Department of Soil, Plant and Food Sciences, Entomological and Zoological Section, University of Bari Aldo Moro, Bari, Italy; ^2^Department of Agronomy, Food, Natural Resources, Animals and Environment, University of Padova, Padova, Italy

**Keywords:** plant feeding mites, mite-host plant interactions, plant-virus vectors, economic importance, hostplant resistance mechanisms, cultivar susceptibility

## Abstract

Eriophyoid mites (Acari Eriophyoidea) are phytophagous arthropods forming intimate relationships with their host plants. These mites are associated with annual and perennial plants including ferns, and are highly specialized with a dominant monophagy. They can be classified in different ecological classes, i.e., vagrant, gall-making and refuge-seeking species. Many of them are major pests and some of them are vectors of plant pathogens. This paper critically reviews the knowledge on eriophyoids of agricultural importance with emphasis on sources for host plant resistance to these mites. The role of species belonging to the family Eriophyidae as vectors of plant viruses is discussed. Eriophyoid-host plant interactions, the susceptibility within selected crops and main host plant tolerance/resistance mechanisms are discussed. Fundamental concepts, subjects, and problems emerged in this review are pointed out and studies are suggested to clarify some controversial points.

## Introduction

Eriophyoids are obligatory plant feeders with unusual morphological, biological and behavioral specialization compared to other Acari (Skoracka et al., 2010). Many of them are major pests of agricultural and ornamental crops, wild plants, grasses, and plants of urban and community forestry but they rarely cause their death (Lindquist et al., 1996). Some of them increase their impact by transmitting plant viruses (de Lillo et al., 2016). Other species are efficient in hampering invasive alien plant species (Smith et al., 2010). Mites of the family Diptilomiopidae are vagrants and have trivial interest. Vice versa, about one third of known species in Phytoptidae and Eriophyidae are gall-making and refuge-seeking, considerably affecting the physiology and production of some relevant crops, even though some vagrants in Eriophyidae can injure severely their hosts (Amrine and de Lillo, pers. database). Crop areas and plant distribution are assuming new geographical borders for climatic changes and for new approaches in agricultural and land management (e.g., requests of cultivars resistant to arid and semi-arid conditions). The bio-ecological features of eriophyoids can affect plants in these “new environments.”

The current paper would update the most recent reviews on the taxon (Ueckermann, 2010) on selected biological and ecological aspects that may explain the intimate relationships between eriophyoids and their host plants. The information gathered here could be devoted to guide future efforts to explore eriophyoid diversity in order to achieve basic, specific and applied goals in plant protection as well as in understanding more general acarological aspects.

## The Eriophyoids as Economically Important Crop Pests

Recent advances on eriophyoids having an economic impact in agriculture and their control deserve to be discussed. Chemical control of eriophyoids has rarely been associated to failures associated with acaricide resistance; this is surprising when compared to worldwide problems encountered in spider mite control (Van Leeuwen and Dermauw, 2016). Nevertheless, a dramatic reduction in pesticide availability has occurred in Europe after the application of the Directive 91/414/EEC and the Regulation (EC) No. 1107/2009 devoted to plant protection products registration (Van Leeuwen et al., 2010) and this tendency is involving also non-EU countries. The reduced number of available acaricides implies potential risks for pesticide resistance and suggests to identify urgently effective non-chemical alternatives. Regarding eriophyoids, most of these studies have been devoted to identify biocontrol agents while research on plant resistance is still lacking or limited to few species.

*Aceria tosichella* Keifer and *A. guerreronis* Keifer appear the most investigated (119 and 107 documents reported in Web of Science Core Collection, respectively, from 1985 to 2018) among the eriophyoids damaging annual and perennial crops, followed by *Aculus schlechtendali* (Nalepa) (65), *Phyllocoptruta oleivora* (Ashmead) (61), *Cecidophyopsis ribis* (Westwood) (50), *A. tulipae* Keifer (50), *Calepitrimerus vitis* (Nalepa) (48), *Aculops lycopersici* (Tryon) (42), *Abacarus hystrix* (Nalepa) (41), *Colomerus vitis* (Pagenstecher) (36) and *Phytoptus avellanae* Nalepa (26). It should be stressed that the number of documents reported for each species in this database does not reflect strictly their economic importance.

The wheat curl mite, *A. tosichella*, infests a wide range of cultivated and wild Poaceae, and genotype MT-1 was able to colonize onion and garlic in laboratory trials (Skoracka et al., 2014a, 2018). It can inflict direct yield loss to wheat, *Triticum aestivum* L., and other cereal crops inducing a syndrome of curled, looped and trapped leaves (Navia et al., 2010; Petanović and Kielkiewicz, 2010b). The main injuriousness of this mite comes from the transmission of wheat streak mosaic virus (WSMV) and wheat mosaic virus (WMoV) which are severe yield-reducing pathogens (Dumón et al., 2013; Richardson et al., 2014; Skoracka et al., 2018). Brome streak mosaic virus (BrSMV) and triticum mosaic virus (TriMV) can also be transmitted (Navia et al., 2013a). This species can house genetically distinct lineages, including generalist ones (Carew et al., 2009; Skoracka et al., 2012, 2013, 2014a,b, 2018). *Aceria tosichella* and the cereal rust mite, *A. hystrix*, can dominate eriophyoid communities associated to wild and cultivated grasses. In a recent survey the first infested the largest number of grass species while the second had the highest incidence on wheat (Kiedrowicz et al., 2014). Host specialization has been widely reported for both species (Skoracka and Dabert, 2010; Miller et al., 2013; Skoracka et al., 2013). Their involvement in virus transmission has promoted research aimed to finding sources of resistance and developing breeding programs (Dhakal et al., 2018). In contrast, studies on factors affecting the incidence of mite infestation and the severity of virus infection are still limited and appropriate control strategies should be substantially improved (Ranabhat et al., 2018).

The dry bulb mite, *A. tulipae*, is an important pest of bulbous plants (e.g., garlic, onion and tulip). Development is optimal at 25°C but eggs can survive at quite large temperature regimes (6–45°C) (Courtin et al., 2000) with clear implications for crop damage. Host range of *A. tulipae* has been recently explored due to its importance for risk assessment and control (Kiedrowicz et al., 2017). A large variation in mite susceptibility among garlic varieties has been reported and the choice of resistant varieties has dramatically reduced *A. tulipae* infestation (Sapakova et al., 2015). Attempts to identify biocontrol agents have suggested a number of candidates (Duarte et al., 2018) but strategies using them should be delineated more in depth.

The tomato russet mite, *A. lycopersici*, tolerates diversified climatic conditions and completes several generations per year causing alterations in leaves, stems and fruits, often up to plant desiccation (Duso et al., 2010). The identification of non-chemical and effective tools to control mite pests on tomato is urgently needed. The use of predatory mites has been largely investigated but the presence of glandular trichomes hinders their settlement. These trichomes can be degraded in plants infested by *A. lycopersici* allowing predatory mites colonization (van Houten et al., 2013). However, tomato russet mite can find refuges in fresh trichome-dense areas of leaves, escaping to predators. The effectiveness of pathogenic fungi against russet mites has been also tested with promising results (Zanolli et al., 2010). Damage intensity varied among tomato cultivars and mite densities, and was lower on wild *Lycopersicon* spp. (Kitamura and Kawai, 2006). These findings were considered useful for breeding tomato resistant cultivars but a significant progress in this field appears still lacking.

The coconut rust mite, *A. guerreronis*, is highly damaging in the coconut production areas (de Lillo and Skoracka, 2010). Patterns in mite invasion, genetic variability in different countries and features of mite-plant interactions suggest that this species moved from its original host (another palm species?) to coconut after the latter was largely cultivated in the Americas and Africa (Navia et al., 2013b). Severe infestations cause fruit distortion and premature dislodging with reduction in crop yield, coconut fiber length and strength, and husk availability (Navia et al., 2013b). The relationship between damage and mite density at different fruit ages has been investigated in Brazil to improve monitoring techniques (Sousa et al., 2017). Since chemical control is expensive, a number of studies have been devoted to biocontrol strategies with promising results (Navia et al., 2013b).

*Aculus schlechtendali* feeds on flowers, fruits and leaves of apple inducing fruit russet, cracking on the cheek and color alterations (Easterbrook, 1996). High infestations cause negative effects on the net CO_2_ exchange and transpiration rates but the impact of mites on apple yield depends on apple cultivar and environmental conditions (Duso et al., 2010). The identification of economic thresholds for the most popular apple cultivars and the adoption of strategies aimed at preserving predatory mite populations are crucial to reduce acaricide use in fruit orchards (Simoni et al., 2018).

The big bud mite, *P. avellanae*, includes two cryptic species based on phylogenetic analyses of mitochondrial cytochrome oxidase subunit I (COI) DNA and the nuclear D2 region of 28S rDNA sequences (Cvrković et al., 2016). The first one, *P. avellanae* s.s., lives in hazelnut buds, causes their increase in size (big buds) and drying. The second one is vagrant and should be named after its morphological characterization and more exhaustive study of bio-ecology. *Phytoptus avellanae* s.s. damages 18–20% of buds in North America and over 50% of buds in South Europe and Middle East (Castagnoli and Oldfield, 1996; Özman and Toros, 1996). The impact of the second species on hazelnut production needs to be ascertained. Biocontrol agents of *P. avellanae* have been largely studied but their management can be difficult due to the side-effects of pesticides used to control other pests. Genetic bases of the susceptibility of hazelnut to big bud mite have been explored (Gantner, 2009) but susceptible cultivars are still preferred by hazelnut industry.

*Phyllocoptruta oleivora* is the most damaging among eriophyoids associated to citrus and frequent acaricide applications are made to reduce its damage (Maoz et al., 2016; Childers et al., 2017). Pesticide contamination and resistance pressed to search for alternatives to acaricides. Most of recent studies deal with biocontrol agents (e.g., Phytoseiidae, Cecidomyiidae, pathogenic fungi) whereas knowledge on cultivar susceptibility/tolerance is quite limited. The pest abundance on sweet oranges can be affected by rootstocks (da Silva et al., 2016). These authors suggested the presence of putative resistance mechanisms to this pest in some genotypes but ad hoc studies are still lacking.

*Cecidophyopsis ribis* causes abnormal growth of buds (big buds) and transmits blackcurrant reversion virus (BRV) that makes plants unproductive (Jones, 2000). Most of commercial blackcurrant cultivars in Western Europe proved to be susceptible to this mite but the impact of damages and reversion diseases was variable among blackcurrant genotypes (Brennan, 1996; Jones et al., 1998). Resistance detected in *Ribes* spp. to *C. ribis* promoted successful breeding programs (see below).

Vineyards can be infested by gall making and vagrant eriophyoids. Among the former, *Co. vitis* has been considered for long time a minor pest. Recent studies pointed out the negative effects of erineum strain of this mite on plant growth and physiology (Javadi Khederi et al., 2014, 2018a). Evidence that erineum strain of *Co. vitis* is involved in the epidemiology of the Grapevine Pinot gris Virus (GPGV) has been provided (Malagnini et al., 2016). Studies on *Co. vitis* aimed at evaluating the impact on grapevine yield and quality, and mite bio-ecology are needed to assess its real pest status and adopt adequate control measures. A number of generalist phytoseiid mites colonizing vineyards prey upon *Co. vitis* (Duso and de Lillo, 1996). Laboratory studies evaluated the effect of a diet based on *Co. vitis* on the demographic parameters of some predatory mites with a potential impact in controlling this pest in realistic conditions (Lorenzon et al., 2012).

*Calepitrimerus vitis* is vagrant and can seriously damage grape growth and yield. A relatively low (<10) number of overwintering females (the so-called deutogynes which are morphologically separated by the spring-summer females known as protogynes) per bud have been associated with leaf and shoot distortions, retarded shoot growth and crop losses (Walton et al., 2007, 2010). Knowledge on the biology and ecology of *Ca. vitis* has been substantially improved in the last two decades (Duffner et al., 2001; Walton et al., 2010; Lee et al., 2015, 2018) allowing to delineate the remarkable potential of this species. Chemical control is often requested because grapevine tissues are susceptible in early spring when populations of natural antagonists are not sufficiently dense to contrast the infestation (Duso et al., 2010). Little is known on cultivar susceptibility to this species and possible sources of resistance have been not identified, yet.

This synopsis focuses on eriophyoid species most cited in the literature. It is worth mentioning that a complex of eriophyoid species can be associated with particular crop systems but they are not enough cited/studied even though their relevant importance in agriculture. As an example, *Aceria sheldoni* (Ewing), *Aculops pelekassi* (Keifer) and *Diptilomiopus floridanus* Craemer & Amrine can infest citrus orchards where *P. oleivora* is dominant (Childers et al., 2017). Other eriophyoids are becoming emerging pests in tropical and subtropical crops. *Aceria litchii* (Kiefer) reduces litchi productivity in Brazil where genetic sources for resistance are under investigation (Arantes et al., 2017). An interesting case study shows that *Aceria mangiferae* Sayed increases the severity of infection caused by the fungal pathogen *Fusarium mangiferae* Britz, M. J. Wingf. & Marasas, in mango (Gamliel-Atinsky et al., 2010). Information on other mite pests infesting major crops in these areas (e.g., rice and banana) is also reported.

## Eriophyoids as Vectors of Plant Viruses

Some species of the family Eriophyidae can transmit plant viruses. They belong to seven genera within the subfamilies Eriophyinae, Phyllocoptinae and Cecidophyinae and have vagrant [*A. hystrix*, *Aceria cajani* Channabasavanna, *Aceria ficus* (Cotte), *A. tosichella, Aculus cercidis* (Hall)], refuge-seeking (*A. tulipae*, *Eriophyes insidiosus* Keifer and Wilson, *Phyllocoptes fructiphilus* Keifer, *P. gracilis* (Nalepa) and gall-making behavior (*C. ribis*, *Co. vitis*, *Eriophyes inaequalis* Wilson and Oldfield, *E. pyri* (Pagenstecher)). They are associated to a single host (i.e.: *A. ficus* on fig; *A. cercidis* on Eastern redbud; *E. inaequalis* on wild bitter cherry), to plant species within the same genus (i.e.: *A. cajani* on *Cajanus* spp.; *C. ribis* on *Ribes* spp.; *Co. vitis* on *Vitis* spp.; *P. fructiphilus* on *Rosa* spp.; *P. gracilis* on *Rubus* spp.) and within the same family (i.e., *A. tosichella* and *A. hystrix* on Poaceae; *E. pyri* on Rosaceae). *Aceria tulipae* is an exception; it has been recorded on *Allium* (Amaryllidaceae), *Tulipa* (Liliaceae) and *Xerophyllum* (Melanthiaceae). This wide range of host plants is quite unusual for Eriophyidae but cryptic species are common. They are currently studied for *A. tosichella*, *A. tulipae*, *A. hystrix* and are presumed for *E. pyri* (Skoracka, 2009; Skoracka and Dabert, 2010; Skoracka et al., 2014a, 2015, 2017, 2018). Phytoptid and diptilomiopid species have never been suspected to be vectors and there are no published studies suggesting their involvement in the virus transmission even though a new emaravirus, blackberry leaf mottle-associated virus (Hassan et al., 2017), may be related to a new diptilomiopids species (Druciarek, pers. comm. 5 Sept., 2018). The application of new techniques and instruments, like Next Generation Sequencing and Illumina, and a more intense collaboration between acarologists, plant physiologists and virologists could clarify some pathosystems and allow detecting new virus-eriophyoid-plant combinations, confirming or rejecting the exclusive interaction between mites of the family Eriophyidae and plant viruses (Skoracka et al., 2018). About two dozens of serious viral diseases of herbaceous and woody plants are associated to eriophyid mites (de Lillo et al., 2016) and new ones are coming (Hassan et al., 2017). An eriophyid-borne virus is transmitted neither by other mites, nematodes or insects nor by more than one eriophyid species (Oldfield and Proeseler, 1996). Also the previously reported vectors of the BRV, *C. ribis* and *C. spicata* Jones (Lemmetty et al., 2004), were recently suggested to belong to a single species by molecular investigation results (Stalažs and Moročko-Bičevska, 2016). The high degree of specificity between mite vectors and viruses (Oldfield and Proeseler, 1996) might depend on the mode of virus acquisition, transmission and inoculation. Biochemical modifications induced by eriophyid saliva injected into the infected plant cells may influence the virus acquisition. Specific helper proteins might mediate the absorption of virus coats on the mite gut. Membrane proteins might translocate viruses through the gut and salivary gland epithelia. All these issues are fairly conjectural and require a histological and biomolecular approach. Similarly, the effects of mite feeding on the plant physiology and biochemistry are far from being clarified.

The impact of *A. tosichella* on crops can benefit from the susceptible volunteer plants and alternative hosts growing at the field edges, fallow fields, along roadsides and natural environments. These plants can represent green bridge refuges for mites and reservoirs for related viruses during non-growing seasons of the primary or elective hosts (Malik et al., 2003; Skoracka and Kuczyński, 2006). Also seeds of wheat, corn or grasses can be a source of WSMV and WMoV even though at low rates (0.07–1.5%) (Jones et al., 2005; Lanoiselet et al., 2008). Spreading of virus-infected seeds in virus-free areas infested by *A. tosichella* could easily start a new infection by means of the mite. In contrast, PPSMV is transmitted only by *A. cajani* on pigeon pea and not by plant sap, seed or dodder (Latha and Doraiswamy, 2008; Maurya et al., 2017) sustaining the management of the virus disease by means of resistant varieties (Pallavi and Ramappa, 2014). The effectiveness of *A. tosichella* in transmitting viruses to the wheat can be influenced by the mite population composition and origin, and it might be related to the genetic mite lineage (Schiffer et al., 2009; Navia et al., 2013b; McMechan et al., 2014; Skoracka et al., 2018). Mite genotypes differ in vectoring ability also for *A. cajani* in transmitting pigeon pea sterility mosaic virus (PPSMV) and *A. hystrix* in spreading ryegrass mosaic virus (Oldfield and Proeseler, 1996; Kumar et al., 2001; Harvey et al., 2005; McMechan et al., 2014).

The effects of the virus infected plants on the biology of the Eriophyidae are poorly known even though a strict co-evolution of the pathosystem may have produced advantages to mites. *Aceria tosichella* and *A. cajani* increase their fecundity rate and density, respectively, on WSMV and PPSMV infected and susceptible plant genotypes (Reddy and Nene, 1980; Kulkarni et al., 2002; Jones et al., 2004; Siriwetwiwat, 2006; Latha and Doraiswamy, 2008; Murugan et al., 2011; Skoracka et al., 2018). Field populations of *P. fructiphilus* were up to 17 times denser on rose rosette disease-symptomatic multiflora rose than on symptomless ones and the virus transmission was more efficient only when the mite was feeding on rapidly growing plant organs, which are more susceptible to the mite and more receptive for virus infection (Epstein and Hill, 1999). Vice versa, a negative effect was found on the reproduction of *A. tosichella* when feeding on plants infected by TriMV, which may be explained by a shorter co-evolutionary mite-virus path, such as a lower nutritional quality of the host or an increase in the production of secondary plant metabolites induced by the virus that are detrimental to the mite (McMechan, 2012). Recent studies showed also a negative differential off-host survival of *A. tosichella* coming from TriMV-infected plants compared with those tested from non-infected and WSMV-infected plants (Wosula et al., 2015). These data suggest that the TriMV-infected plants decreased *A. tosichella* response to environmental stress factors, like the absence of the elective host plant.

## Molecular and Biochemical Eriophyoid-Plant Interactions

Host plants genotypes, plant age, mite’s life style, species and strains are crucial in determining the type of plant changes induced by the eriophyoids (Petanović and Kielkiewicz, 2010a,b; Skoracka et al., 2010; Chakrabarti et al., 2011; Cvrković, 2012).

Morphological, biochemical and physiological responses of plants to eriophyoids are still inadequately studied (Petanović and Kielkiewicz, 2010a,b; Chetverikov et al., 2015). A relevant role has to be played by the salivary secretions injected into the plant tissues, whereas the mechanical action causes only accumulation of chitosan and callose at the feeding site as a wound response of the plant (Petanović and Kielkiewicz, 2010a,b). Saliva of piercing and sucking insects is able to degrade cell walls and middle lamella suggesting cellulolytic and pectinolytic enzyme involvement. Studies on insects suggested a potential involvement of the oligosaccharides produced from pectin components of plant cells as a wound messenger in the induction of plant defense responses (Ryan, 2000; Walling, 2000; Gatehouse, 2002). Polygalacturonase (a pectinase) and cellulase activity was documented in saliva of the gall-making *Aceria caulobia* (Nalepa) (Monfreda and Spagnuolo, 2004; Monfreda and de Lillo, 2010). How the salivary secretions of eriophyoids interact with and disrupt the constitutive plant defenses has to be explained, yet, even though potentiality may come from a secretome investigations such as made in spider mites (Villarroel et al., 2016). The presence of other enzymes into the eriophyoid saliva is expected. Techniques and protocols for getting saliva from these tiny mites should be improved in order to routinely collect saliva. Preliminary bioassays have been carried out on off-host eriophyoids treated with some neurotransmitters which acted as stimulators of salivary secretions making the collection more easily reproducible (Monfreda and de Lillo, 2010). Collected saliva might be processed by the most advanced instruments (e.g., Spectrophotometry, HeadSpace Gas Chromatography, Gas Chromatography/Mass Spectrometry, etc.) and analytical techniques (e.g., MALDI) which have lower detection limits.

Vagrant mites can be found on all green surfaces of the plants. Their low population densities do not cause damage, but high population densities may be responsible of phytotoxemias and non-distortive alterations. Injured epidermal cells may collapse and die soon after piercing (Petanović and Kielkiewicz, 2010a,b). A repeated attack to close portions of epidermal cells induces typical leaf surface alterations (e.g., bronzing, russeting, silvering, discoloration). In many other cases, cells adjacent to the pierced ones may respond to mite injury primarily with the accumulation of higher amounts of lignin-like compounds and with cell walls thickening that may involve in a spongy parenchyma (Petanović and Kielkiewicz, 2010a). *Aculops lycopersici*, a typical vagrant species, induces hypersensitive reactions in the pierced epidermal cells and accumulation of lignin-like compounds in the walls of the closest intact cells (Royalty and Perring, 1988). Short feeding time of *A. lycopersici* on four-leaf tomato plants induced stronger lipoxygenase (LOX) and peroxidase (POD) responses on leaflets of the same leaf (leaf-systemic spatial response) and a strong increase in the peroxidase (POD) activity on leaflets relatively far from the infested leaves (plant-systemic spatial response) (Stout et al., 1996). On tomato, the effect of *A. lycopersici* infestation on the defense regulated by the jasmonic acid (JA) and the salicylic acid (SA) pathways was studied in detail (Glas et al., 2014). Tomato russet mite suppressed the JA downstream defense responses and this was independent from the production and accumulation of salicylic acid (SA), which is the natural antagonist of JA. They also evaluated the effect of the contemporary presence of *A. lycopersici* and *Tetranychus urticae* Koch, showing that in this condition large colonies of the spider mite developed while population growth of russet mites was inhibited. These phenomena might be related to the altered balance in the plant defense chemicals and accumulation of SA. Furthermore, the authors found that SA was not systemically spread to the whole leaflets and its higher amounts were detected near the mite feeding sites. The accumulation of SA induced by *A. lycopersici* infestation seemed to inhibit the growth of *Pseudomonas syringae* pv. *tomato* DC3000, suggesting a role in limiting the development of secondary infections by pathogens, which could make the substrate unsuitable for *A. lycopersici*. Population densities of *A. lycopersici* can increase faster also on drought-stressed tomato plants (Ximenez-Embun et al., 2017). Several biochemical changes were detected in tomato under mite attack and/or drought stress periods. Levels of total proteins along with some free amino acids and free sugar increased only when mite infestation and drought were combined. It should be emphasized that free sugars act as feeding stimulants for herbivorous mites. *Aculops lycopersici* induced an increase in JA, in its precursor (OPDA) and bioactive form (JA-Ile). When drought was combined with mite infestation, OPDA and SA increased further in contrast with JA accumulation. Mite infestation downregulated the expression of some JA-marker genes, while transcript accumulation for the SA-marker gene increased. Mite infestation alone increased the activity of cysteine protease inhibitors, PPO and POD. Drought contrasted the accumulation of POD and JA induced by *A. lycopersici*, while it synergized the accumulation of SA. These effects have clear implications in the framework of climate change, which are expected to increase the periods of drought. Ximenez-Embun et al. (2017) proved that on drought-stressed tomato plants, *A. lycopersici* can found favorable conditions: high nutritional value and reduced levels of induced defenses (i.e., transcript level of JA-associated genes). Changes in phenolic compound concentrations were observed on olive fruits infested by *Aculus olearius* Castagnoli. The amount of tyrosol and vanillic acid decreased and increased, respectively, and fruits with lower concentrations of tyrosol were more susceptible to mite damage (Çetin et al., 2011). Changes in volatile organic compounds emitted by plants infested by *A. lycopersici* have also been reported and may be related to changes in defense pathways triggered by this vagrant mite (Takayama et al., 2013). Little is known on the molecular mechanisms underlying these interactions.

Refuge seeking mites can exploit preexisting shelters in needles and leaf sheets, in buds and between bulb scales. Gall-making mites induce abnormalities in plant tissues in form of leaf curls, erinea, pouched galls, blisters, witches’ brooms, big buds, organ deformations (Chetverikov et al., 2015), which create refuges as well as foraging sites. *Hibiscus vitifolius* L. infested by the gall mite *Acalitus hibisci* Mondal & Chakrabarti showed an increase in leaf hairs density with implications for mite success (Chakrabarti et al., 2001). All these abnormalities are usually induced on young, in-growth and tender tissues of the plant apart from the roots. Eriophyoids are supposed to synthesize and secrete chemicals which cause local changes in the metabolism and balance of plant hormones, as well as can trigger biochemical chain reactions responsible for induction and growth of plant abnormalities (Chetverikov et al., 2015). Saliva of *A. caulobia*, inducing stem galls on shrubby seablight, was proved to have indolacetic acid (IAA) and cytokinin-like activities through wheat-coleoptile and radish-cotyledon growth bioassays (de Lillo and Monfreda, 2004). But little is known on the nature of this activity and if saliva contains plant growth regulators or other chemicals (proteins and peptides) which can alter the synthesis of plant hormones in the infested cells. In any case, pouched galls result from the de-differentiation of the adjacent parenchymal cells in a meristematic tissue, from the proliferation of epidermal and parenchymal cells, and from the protrusion of neoplasic structures from the organ surfaces (Petanović and Kielkiewicz, 2010a; Chetverikov et al., 2015). These histological reactions are in accordance with the changes induced by *Fragariocoptes setiger* (Nalepa) in the expression of transcription factors involved in meristem activity, plant hormone secretion, cell mitosis and adaxial–abaxial galled leaf polarity during gall morphogenesis on *Fragaria viridis* Weston (Paponova et al., 2018), demonstrating a complex biochemical network which has to locally restrain the mite.

Practically useless fine details are available on mite-plant interactions for gall-making species inducing other plant abnormalities apart pouched galls. Recently, the erineum strain of *Co. vitis* was demonstrated to decrease the leaf area, the plant height, internode length, and chlorophyll content, and to increase the leaf fresh weight as the main effect of hypertrophy and hyperplasia of epidermal and mesophyll cells of the erinea (Javadi Khederi et al., 2014, 2018b,c). The content of chlorophyll decreased also in leaf galls induced by *A. hibisci* on *H. vitifolius*, whereas the amount of carotene was increasing in comparison to the healthy tissues (Chakrabarti et al., 1999). Changes in chemical composition of edge-rolled and erineal leaves were verified on *Tilia* spp. infested by *Phytoptus tetratrichus* (Nalepa) (Kielkiewicz et al., 2011; Soika et al., 2017). High number of starch grains was assessed within the nutritive and hypertrophied parenchyma cells as well as accumulation of antioxidant flavonols, anthocyanins and tannins was detected on densely infested leaves. Starch can suggest a high metabolic activity during abnormality formation. The increase of flavonoid levels, whose protective action from reactive oxygen species (ROS) is well-know, might be the result of an induced defense action triggered by mite saliva injection, even though histochemical analysis evidenced the presence of poliphenolic compounds also inside the mite body (Kielkiewicz et al., 2011). Silencing polyphenol oxidase (PPO) activity, at the early stage of gall formation, might influence the monophenol flavonols stressing the mite-plant interactions and given further evidences of the defense mechanism. But also the regulation of the expression of other factors related to anthocyanins and tannins might help in understanding the gall induction and development mechanisms.

Apart vagrant (e.g., *P. oleivora* and *A. lycopersici*) and gall-making (e.g., *Aceria sheldoni* [Ewing]) species that damage fruits, eriophyoid mites can impact indirectly plant productivity. The physiological processes of transpiration and photosynthesis can be negatively influenced in different plant-mite species combinations involving vagrants (Daud et al., 2012) and gall-makers (Javadi Khederi et al., 2018b). These effects can have also large scale and long term impacts on community forestry where gall inducing mites have proven to be a major driver of age-related declines in tree performance and patterns of net primary productivity (Patankar et al., 2011).

## Plant Resistance Mechanisms and Selection

**FIGURE 1:**
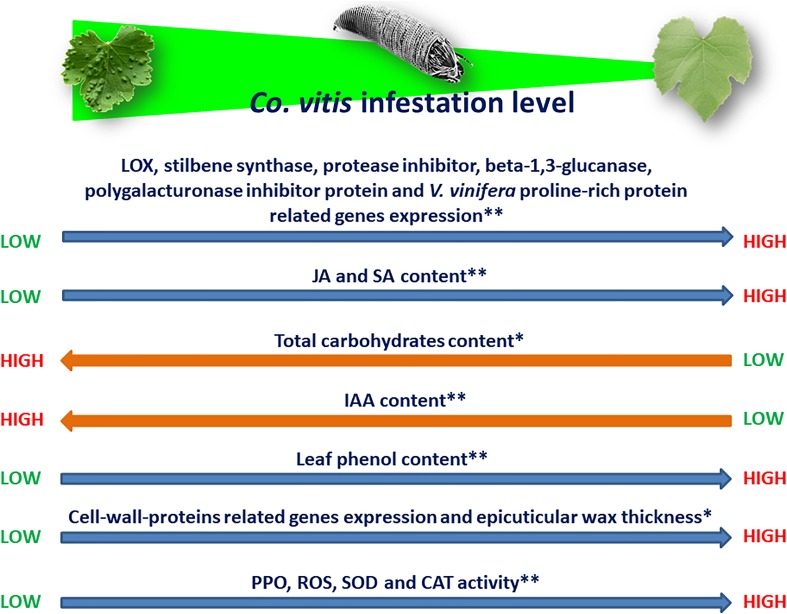
Direction of interactions between *Co. vitis* infestation level and different grapevine’s traits. See text for acronyms definition. ^∗^Constitutive traits; ^∗∗^Induced traits.

Knowledge on the physiological changes in infested plants, their genetic basis and heritability are fundamental for the implementation of resistance/tolerance of the host plants (Mitchell et al., 2016; Stenberg and Muola, 2017). The effects induced by the eriophyoid feeding on their hosts may not be always forecasted. Some of them showed a notable variability depending on mite and plant genotype, mite density, feeding period, plant age and environmental conditions (Royalty and Perring, 1996; Westphal and Manson, 1996; Duso et al., 2008; Petanović and Kielkiewicz, 2010a,b). Resistant plants respond to their feeders mainly by changes in the expression of genes related to defense. For example, the increase of the ratio between the total phenolic compounds (feeding inhibitors) and the total carbohydrates/proteins (feeding stimulators) in leaves of blackberry infested by *Epitrimerus gibbosus* (Nalepa) were suggested to limit mite success (Shi and Tomczyk, 2001). Similarly, *Co. vitis* appeared not thriving on grapevine cultivars with increasead leaf phenol content during mite infestation (Javadi Khederi et al., 2014; Figure 1). The levels of epicuticular wax thickness and leaf carbohydrate content were negatively correlated with the mite density and the incidence of erinea (Javadi Khederi et al., 2014; Figure 1). *Colomerus vitis*, like other gall-making eriophyoids, redirects the development of pierced cells and their closest ones. Consequently, the plant’s physiology, and the shape and size of infested growing organs are modified (Petanović and Kielkiewicz, 2010a,b; Javadi Khederi et al., 2014, 2018b,c,d). Little is known on the expression of defense genes among different genotypes. Six plant marker genes [LOX, stilbene synthase, protease inhibitor, beta-1,3-glucanase, polygalacturonase inhibitor protein and *V. vinifera* proline-rich protein of three Iranian grapevine cultivars displayed differential responses shortly after *Co. vitis* infestation (Javadi Khederi et al., 2018a; Figure 1)]. Lower expression of the above mentioned genes was always observed in the susceptible cultivar (Ghalati), whereas the genes for the highly (Atabaki) and the moderately resistant (Muscat Gordo) cultivars were quite differently regulated during that time. The highest upregulated expression of LOX (ethylene-associated gene) in the highly resistant cultivar (Atabaki) is in accordance with the pattern of the same gene in roots of grapevine resistant to the plant-parasitic hemipteran *Daktulosphaira vitifoliae* Fitch (Phylloxeridae) (Blank et al., 2009). The rate of ethylene synthesis in pierced tissues is influenced by the salivary indole-3-acetic-acid (IAA) amount injected or accumulated in them, and ethylene may be involved in incompatible mite-plant interactions (Javadi Khederi et al., 2018a; Figure 1).

The involvement of IAA and phenolic compounds in gall making mite-plant interactions was pointed out in *Co. vitis* (Javadi Khederi et al., 2018c), *Aceria cherianii* (Massee), *A. cernuus* (Massee) (Balasubramanian and Purushothaman, 1972a,b; Tandon and Arya, 1980; Tandon, 1985), *F. setiger* (Paponova et al., 2018) and was recently reviewed (Chetverikov et al., 2015). Density of *Co. vitis* on a highly susceptible cultivar (Sezdang) was positively correlated with IAA content of the infested leaves and mites appeared to benefit when leaf IAA increased more than JA and SA, which were negatively correlated with infestation levels (Javadi Khederi et al., 2018c; Figure 1). The putative presence of IAA or related compounds was also demonstrated in salivary secretions of *A. caulobia* (de Lillo and Monfreda, 2004). Also, genes encoding cell-wall-proteins (polygalacturonase inhibitor protein and *V. vinifera* proline-rich protein 1) were upregulated in the grapevine cultivar highly resistant to *Co. vitis* (Atabaki) (Javadi Khederi et al., 2018a; Figure 1). Both encoded proteins oppose the degradation of cell wall architecture caused by the feeder saliva. Hydrogen peroxide and the activity of PPO, superoxide dismutase (SOD) and catalase (CAT) enzymes were found to explain the responses of grape cultivars with a different susceptibility to *Co. vitis* infestation (Javadi Khederi et al., 2018d; Figure 1). Hydrogen peroxide is one of the most common ROS induced by environmental stress and was highly and negatively correlated with the mite infestation. SOD and CAT activity was mostly higher in the least susceptible cultivars (Figure 1). They are strictly related to each other and both detoxify the overproduction of ROS: SOD transforms the highly toxic free radical superoxide in oxygen and in the less toxic hydrogen peroxide (Petkau et al., 1975); CAT degrades hydrogen peroxide in water (Storey, 1996). Particularly, PPO displayed a highly negative correlation with the infestation. Its high activity on cultivars resistant to *Co. vitis* might be related to the increase of phenols, decrease of the nutritional quality, lignification and hypersensitive responses of the injured tissues (Thipyapong and Steffens, 1997; Mayer, 2006).

The genetic basis of *A. schlechtendali* resistance in apple was investigated in Switzerland, where a number of different genotypes were monitored for mite susceptibility. A Quantitative Trait locus (QTL) analysis was carried out using data available for F1 progeny plants of the cultivars “Fiesta × Discovery.” Two QTLs for *A. schlechtendali* resistance on linkage group 7 of “Fiesta” were identified. The identification of a Simple Sequence Repeat (SSR) marker associated to one of the QTLs was considered a first step for the evaluation of resistance to rust mites and the breeding of resistant apple cultivars (Stoeckli et al., 2009). The functional importance of these markers was not fully defined. The “Fiesta” × “Discovery” apple progeny is characterized by adequate fruit firmness, sugar content and acidity but the infestation of various apple pests (excluded *A. schlechtendali*) was positively correlated with apple high quality features. This phenomenon has been explained as a trade-off between resource allocation to defensive secondary metabolites or to fruit (Stoeckli et al., 2011). This study stressed the need to consider pest resistance when breeding for high quality apple cultivars and advantages using genetic markers for fruit quality and pest resistance.

The most important source of constitutive resistance to *P. avellanae* was represented by the content of secondary metabolites, including tannins (Gantner, 2009). Also essential oil components occurring in the buds of differently susceptible cultivars showed allelopathic effects, like in the less affected Mogulus cultivar, in which nerol, α-campholenol, methyl salicylate, spatulenol, β-caryophylene and δ-cadinene were at higher concentrations compared to the most affected Barra cultivar (Gantner and Najda, 2013). A role of these compounds in deterring mites from feeding was suggested. Differences in varietal susceptibility have also been widely observed for coconut plants against *A. guerreronis* and the physical characteristics of perianth and fruits have been suggested to be involved in plant resistance and reduced plant susceptibility (Navia et al., 2013a). Molecular markers associated with plant resistance have been identified in India (Shalini et al., 2007). However, little is known about the outcome of breeding programs in the selection of mite-resistant coconuts. Experiment design for the evaluation of the assessment of the biological and behavioral responses in mites to different varieties (Stenberg and Muola, 2017) could help in defining future directions in breeding programs.

Currently, breeding program for the selection of resistant plant species have been mainly addressed to viruses and their eriophyid vectors like wheat against *A. tosichella*, pigeon pea and *A. cajani*, black and red currants against *C. ribis* (Jones, 2002; Kumar et al., 2005; Van Leeuwen et al., 2010; Petanović et al., 2017; Chuang et al., 2017; Skoracka et al., 2018) and a few others.

For example, resistance detected in *Ribes* species to *C. ribis* promoted breeding programs focused on Ce and P genes originating from *R. uva-crispa* L. and *R. nigrum* L. var. *sibiricum*, respectively (Anderson, 1971; Knight et al., 1974; Keep et al., 1982; Brennan et al., 2009; Sasnauskas et al., 2009). Eriophyids cannot penetrate the buds of Ce-genotypes, while they cannot survive for long periods of time in buds of P-genotypes. They can transmit BRV to these genotypes but at a low incidence (Jones et al., 1998; Mitchell et al., 2011). Molecular markers for Ce and P genes have been developed (Brennan et al., 2009; Mazeikiene et al., 2012) and recently applied to investigate the origin of resistance to mites in a number of *Ribes* species assessing the inheritance of resistance in genotypes obtained by interspecific hybridization (Mazeikiene et al., 2017). Very recently, Stalažs and Moročko-Bičevska (2016) identified a complex of *Cecidophyopsis* species (based on phylogenetic analyses), besides *C. ribis*, on cultivated and wild blackcurrant in Latvia and some of them could play a significant role in damaging currants. According to these results, future breeding programs for host resistance to Eriophyids mites should also consider other *Cecidophyopsis* species other than *C. ribis*.

The utilization of pest resistant plants is an easy-to-apply strategy, compatible with other control means and ecological friendly for the natural enemies of the target pest. The development of programs for the exploitation of plant defense mechanisms or other finer strategies involving application of non-transgenic methods and genome editing cannot be allowed for the eriophyoids, yet. Most of the research on host resistance to eriophyoid mites has been focused on reducing the potential for pest population development, focusing mostly on antibiosis while scarce attention has been posed on tolerance (Sperotto et al., 2018). Future studies should be aimed at the identification of biological mechanisms underlying the maintenance of crop productivity independently of eriophyoid infestation. It has been suggested that the use of tolerant varieties, having no effect on pest biology are expected to be a more stable and long-lasting strategy (Peterson et al., 2017; Sperotto et al., 2018).

Research on *A. tosichella* may provide an example where identification on tolerant varieties could have interesting perspectives. On this topic, research initially focused on identifying varieties with low trichomes density and length that was thought to reduce landing efficiency of the mites, but breeding program for these traits were never initiated (Navia et al., 2013a). More research effort has been aimed at the identification of genes bearing resistance, mainly antibiosis (Richardson et al., 2014). These were identified in common wheat and related species (Li et al., 2002; Harvey et al., 2003; Navia et al., 2013a; Aguirre-Rojas et al., 2017). Based on these results breeding programs were implemented resulting in commercial wheat cultivars (Martin et al., 1983; Carver et al., 2016). However, different mite population were found to overcome resistance genes in commercial varieties with pest population build-up and this can depend on adaptation of different mite biotypes (Malik et al., 2003; Hein et al., 2012; see Skoracka et al., 2018 for details). In some cases the tolerance has also been evaluated finding varieties which were able to tolerate the wheat curl mite infestation (Carrera et al., 2012). This can provide the basis for breeding program for commercial exploitation of tolerant varieties that could be coupled with plant resistance to viruses for future mite and virus sustainable management options (Carrera et al., 2012).

## Future Directions

Knowledge on mite-plant relationships with or without virus interactions appears to be scanty and fragmented and it may depend on tiny sizes of mites, difficulties with their manipulation especially for suitable feeding substrate and micro-environmental conditions, low numbers of specialists on eriophyoids, few non-taxonomic studies, etc.

Composition of the plant sap ingested by eriophyoids is unknown and it might be supposed that plant defense compounds may interact with saliva within the cells and may interfere with the physiological processes into the mite gut. Transcriptome and proteome analysis addressed to the salivary compounds appear to give a relevant support in understating abnormality inducing processes in gall-making species as well as for the most intimate interactions with the most noxious vagrant and refuge-seeking species in the attempt to identify potential silencing genes. The recent achievements on dsRNA delivery methods also on mites (Suzuki et al., 2017) may make these studies easier, but eriophyoid transcriptome and genome still need to be processed overcoming technical problems related mainly with their tiny size. The key role of IAA and cytokinins in gall-induction needs to be detailed, as well as sustained by explaining the role of the other phytohormones. Research on stimulating the collection of saliva should be supported in order to achieve a protocol for getting a more purified solution to be analyzed with more sensitive instruments developed in the recent years. The approaches used in other mite-plant models could constitute a framework for future studies. In particular, transcriptomic and proteomic on *T. urticae*-plant interactions with shaded light on the interplay between host plants and mite can allow the study from the protein constituents of mite saliva and their functions (Jonckheere et al., 2016, 2018; Villarroel et al., 2016; Rioja et al., 2017) to the responses of plants to the mite attack (Santamaria et al., 2013, 2017; Bensoussan et al., 2016; Diaz-Mendoza et al., 2017; Liu et al., 2017). Mite-plant interactions can be influenced by the environment through its effect on the plant status, i.e., drought stress (Ximenez-Embun et al., 2017; Santamaria et al., 2018), and this aspect appear of particular importance for eriophyoids. Potentially, the results could be used to exploit genomic information and new technologies to accelerate breeding program for resistance and tolerance in crops infested by eriophyoids (Salinas et al., 2013; Martel et al., 2015; Díaz-Riquelme et al., 2016; Gascuel et al., 2017; Karkute et al., 2017; di Donato et al., 2018; Haque et al., 2018). One of the main limitation for the implementation of these types of studies on eriophyoids is the absence of efficient mass rearing methods on artificial substrates (Cazaux et al., 2014; Jonckheere et al., 2016) for which cooperation between plant and mite scientists is needed for its development.

This will require additional knowledge on the gut anatomy, cellular organization and physiology, which can explain the interactions with the ingested chemicals as well as the detoxification of plant metabolites, the protective physiology of the gut cells, and the inactivation of enzyme inhibitors. Histological and microscopy techniques combined with the study of the expression pattern of digesting/detoxifying genes may present a valuable research line.

Finally, the microbiome associated to the eriophyoids and its effect on plant-mite interaction is a field completely unexplored. The idea that eriophyoids might transmit gall-inducing viruses or bacteria remains a fascinating and unverified hypothesis till now (Chetverikov et al., 2015), even though the new biotechnologies could assist in looking for entities with very small DNA and RNA. Whether individuals of different generations and morphs (protogynes *versus* deutogynes) have the same efficiency and mechanisms in inducing galls is still an open question.

## Author Contributions

All authors listed have made a substantial, direct and intellectual contribution to the work, and approved it for publication.

## Conflict of Interest Statement

The authors declare that the research was conducted in the absence of any commercial or financial relationships that could be construed as a potential conflict of interest.

## References

[B1] Aguirre-Rojas L. M. Khalaf L. K. Garcés-Carrera S. Sinha D. K. Chuang W.-P. Michael Smith C. (2017). Resistance to wheat curl mite in arthropod-resistant rye-wheat translocation lines. *Agronomy* 7 74 10.3390/agronomy7040074

[B2] Anderson M. M. (1971). Resistance to gall mite (*Phytoptus ribis* Nal.) in the *Eucoreosma* section of *Ribes*. *Euphytica* 20 422–426. 10.1007/BF00035668

[B3] Arantes R. F. De Andrade D. J. Amaral I. Martins A. B. G. (2017). Evaluation of litchi varieties seeking sources resistant to aceria *litchi* mite. *Rev. Bras. Frut* 39 1–7. 10.1590/0100-29452017816

[B4] Balasubramanian M. Purushothaman D. (1972a). Indole acetic acid in the eriophyid mite gall on *Pongamia glabra* vent. caused by *Eriophyes cheriani* massee (Eriophyidae: Acarina). *Labdev. J. Sci. Technol.* 10 172–173.

[B5] Balasubramanian M. Purushothaman D. (1972b). Phenols in healthy and galled leaves of *Pongamia glabra* vent. caused by an eriophyid mite, *Eriophyes cheriani* Massee (Eriophyidae: Acarina). *Indian J. Exp. Biol.* 10 394–395.

[B6] Bensoussan N. Santamaria M. E. Zhurov V. Diaz I. Grbić M. Grbić V. (2016). Plant-herbivore interaction: dissection of the cellular pattern of *Tetranychus urticae* feeding on the host plant. *Front. Plant Sci.* 7:1105. 10.3389/fpls.2016.01105 27512397 PMC4961969

[B7] Blank L. Wolf T. Eimert K. Schröder M. B. (2009). Differential gene expression during hypersensitive response in *Phylloxera*-resistant rootstock ‘Börner’ using custom oligonucleotide arrays. *J. Plant Interact.* 4 261–269. 10.1080/17429140903254697

[B8] Brennan R. M. (1996). “Currants and gooseberries,” in *Fruit Breeding*, Vol. II, *Vine and Small Fruits Crops*, eds Janick J. Moore J. N. (New York, NY: Wiley), 191–295.

[B9] Brennan R. Jorgensen L. Gordon S. Loades K. Hackett C. Russell J. (2009). The development of a PCR-based marker linked to resistance to the blackcurrant gall mite (*Cecidophyopsis ribis* Acari: Eriophyidae). *Theor. Appl. Genet.* 118 205–211. 10.1007/s00122-008-0889-x 18813905

[B10] Carew M. Schiffer M. Umina P. Weeks A. Hoffmann A. (2009). Molecular markers indicate that the wheat curl mite, Aceria *tosichella* keifer, may represent a species complex in Australia. *Bull. Entomol. Res.* 99 479–486. 10.1017/S0007485308006512 19224660

[B11] Carrera S. G. Davis H. Aguirre-Rojas L. Murugan M. Mike Smith C. (2012). Multiple categories of resistance to wheat curl mite (Acari: Eriophyidae) expressed in accessions of *Aegilops tauschii*. *J. Econ. Entomol.* 105 2180–2186. 10.1603/EC12252 23356085

[B12] Carver B. F. Smith C. M. Chuang W.-P. Hunger R. M. Edwards J. T. Yan L. (2016). Registration of OK05312, a high-yielding hard winter wheat donor of Cmc4 for wheat curl mite resistance. *J. Plant Regist.* 10 75–79. 10.3198/jpr2015.04.0026crg

[B13] Castagnoli M. Oldfield G. N. (1996). “Other fruit trees and nut trees,” in *Eriophyoid Mites-Their Biology, Natural Enemies and Control*, eds Lindquist E. E. Sabelis M. W. Bruin J. (Amsterdam: Elsevier Science B.V.), 543–559. 10.3791/51738

[B14] Cazaux M. Navarro M. Bruinsma K. A. Zhurov V. Negrave T. Van Leeuwen T. (2014). Application of two-spotted spider mite *Tetranychus urticae* for plant-pest interaction studies. *J. Vis. Exp.* 89:e51738. 10.3791/51738 25046103 PMC4211727

[B15] Çetin H. Arslan D. Musa Özcan M. (2011). Influence of eriophyid mites *Aculus olearius* castagnoli and aceria oleae (Nalepa) (Acarina: Eriophyidae) on some physical and chemical characteristics of ayvalik variety olive fruit. *J. Sci. Food Agric.* 91 498–504. 10.1002/jsfa.4212 21218484

[B16] Chakrabarti S. Chakrabarti S. Chakrabarti S. (1999). Effect of *Acalitus hibisci* (Eriophyoidea) infestation on photosynthetic pigments of *Hibiscus vitifolius*. *J. Acarol.* 14 49–51.

[B17] Chakrabarti S. Chakrabarti S. Chakrabarti S. (2001). Effect of infestation of *Acalitus hibisci* (Eriophyoidea: Eriophyidae), a gall forming mite on age and hairs of leaves of *Hibiscus vitifolius*. *Acarologia* 41 313–316.

[B18] Chakrabarti S. Chakrabarti S. Chakrabarti S. (2011). Changes in leaf chemistry of *Hibiscus vitifolius* L. due to gall induction by an eriophyoid mite, *Acalitus hibisci* mondal and chakrabarti. *Proc. Nat. Acad. Sci. India. Sect. B* 81 190–197.

[B19] Chetverikov P. E. Vishyakov A. E. Doducva I. T. Osipova M. A. Sukhareva S. I. Shavarda A. L. (2015). Gallogenesis induced by eriophyoids (Acariformes: Eriophyoidea). *Parazitologiya* 49 365–375. 26946826

[B20] Childers C. C. Rogers M. E. Ebert T. A. Achor D. S. (2017). *Diptilomiopus floridanus* (Acari: Eriophyoidea: Diptilomiopidae): its distribution and relative abundance with other eriophyoid species on dooryard, varietal block, and commercial citrus in florida. *Flo. Entomol.* 100 325–333. 10.1653/024.100.0230

[B21] Chuang W. P. Rojas L. M. A. Khalaf L. K. Zhang G. Fritz A. K. Whitfield A. E. (2017). Wheat genotypes with combined resistance to wheat curl mite, wheat streak mosaic virus, wheat mosaic virus, and triticum mosaic virus. *J. Econ. Entomol.* 110 711–718. 10.1093/jee/tow255 28087646

[B22] Courtin O. Fauvel G. Leclant F. (2000). Temperature and relative humidity effects on egg and nymphal development of *Aceria tulipae* (K.) (Acari : Eriophyidae) on garlic leaves (Allium sativum L.). *Ann. Appl. Biol.* 137 207–211. 10.1111/j.1744-7348.2000.tb00061.x

[B23] Cvrković T. Chetverikov P. Vidović B. Petanović R. (2012). “Molecular Analysis of COI mtDNA in *Phytoptus* (Phytoptidae) and *Eriophyes* (Eriophyidae) species associated with galls of *Tilia* spp. (Tiliaceae): preliminary results,” in *Proceedings of the International Symposium on Current Trends in Plant Protection*, (Belgrade, Serbia).

[B24] Cvrković T. Chetverikov P. Vidovic B. Petanović R. (2016). Cryptic speciation within *Phytoptus avellanae* s.l. (Eriophyoidea: Phytoptidae) revealed by molecular data and observations on molting Tegonotus-like nymphs. *Exp. Appl. Acarol.* 68 83–96. 10.1007/s10493-015-9981-5 26530992

[B25] da Silva R. R. Teodoro A. V. Vasconcelos J. F. Martins C. R. Soares W. D. de Carvalho H. W. L. (2016). Citrus rootstocks influence the population densities of pest mites. *Ciencia Rural* 46 1–6. 10.1590/0103-8478cr20150486

[B26] Díaz-Riquelme J. Zhurov V. Rioja C. Pérez-Moreno I. Torres-Pérez R. Grimplet J. (2016). Comparative genome-wide transcriptome analysis of vitis vinifera responses to adapted and non-adapted strains of two-spotted spider mite, tetranyhus urticae. *BMC Genomics* 17:74. 10.1186/s12864-016-2401-3 26801623 PMC4724079

[B27] Daud R. D. de Conforto E. C. Feres R. J. F. (2012). Changes in leaf physiology caused by *Calacarus heveae* (Acari, Eriophyidae) on rubber tree. *Exp. Appl. Acarol.* 57 127–137. 10.1007/s10493-012-9552-y 22527832

[B28] de Lillo E. Monfreda R. (2004). “Salivary secretions” of eriophyoids (Acari: Eriophyoidea): first results of an experimental model. *Exp. Appl. Acarol.* 34 291–306. 15651526 10.1007/s10493-004-0267-6

[B29] de Lillo E. Skoracka A. (2010). What’s “cool”on eriophyoid mites? *Exp. Appl. Acarol.* 51 3–30. 10.1007/s10493-009-9297-4 19760102

[B30] de Lillo E. Valenzano D. Saldarelli P. (2016). Attuali conoscenze degli eriofioidei vettori di virus. *Atti Acc. Naz. It. Entomol.* 63 113–121.

[B31] Dhakal S. Tan C. T. Anderson V. Yu H. J. Fuentealba M. P. Rudd J. C. (2018). Mapping and KASP marker development for wheat curl mite resistance in “TAM 112” wheat using linkage and association analysis. *Mol. Breed.* 38:119 10.1007/s11032-018-0879-x

[B32] di Donato A. Filippone E. Ercolano M. R. Frusciante L. (2018). Genome sequencing of ancient plant remains: findings, uses and potential applications for the study and improvement of modern crops. *Front. Plant Sci.* 9:441. 10.3389/fpls.2018.00441 29719544 PMC5914272

[B33] Diaz-Mendoza M. Velasco-Arroyo B. Santamaria M. E. Diaz I. Martinez M. (2017). HvPap-1 C1A protease participates differentially in the barley response to a pathogen and an herbivore. *Front. Plant Sci.* 8:1585. 10.3389/fpls.2017.01585 28955371 PMC5601043

[B34] Duarte A. F. da Cunha U. S. de Moraes G. J. (2018). Suitability of edaphic arthropods as prey for proctolaelaps bickleyi and cosmolaelaps brevistilis (Acari: Mesostigmata: Melicharidae, Laelapidae) under laboratory conditions. *Exp. Appl. Acarol.* 74 275–282. 10.1007/s10493-018-0229-z 29468347

[B35] Duffner K. Schruft G. Guggenheim R. (2001). Passive dispersal of the grape rust mite *Calepitrimerus vitis* nalepa 1905: (Acari, Eriophyoidea) in vineyards. *J. Pest Sci.* 74 1–6. 10.1046/j.1439-0280.2001.01001.x

[B36] Dumón A. D. Argüello Caro E. B. Alemandri V. Mattio M. F. Donaire G. Alberione E. (2013). Performance of different wheat cultivars to wheat streak mosaic virus (WSMV) and high plains virus (HPV) by artificial infection with the vector *Aceria tosichella* keifer under field conditions. *Rev. Invest. Agropec.* 39 67–76.

[B37] Duso C. Castagnoli M. Simoni S. Angeli G. (2008). “The impact of eriophyoids on crops: new and old case studies,” in *Integrative Acarology. Proceedings of 6th European Congress*, eds Bertrand M. Kreiter S., K. McCoy, A. Migeon, M. Navajas, M. S. Tixier, (Montpellier: European Association of Acarolgists), 317–324.

[B38] Duso C. Castagnoli M. Simoni S. Angeli G. (2010). The impact of eriophyoids on crops: recent issues on *Aculus schlechtendali*, *Calepitrimerus vitis* and *Aculops lycopersici*. *Exp. Appl. Acarol.* 51 151–168. 10.1007/s10493-009-9300-0 19757100

[B39] Duso C. de Lillo E. (1996). “Grape,” in *Eriophyoid Mites-Their Biology, Natural Enemies and Control*, eds Lindquist E. E. Sabelis M. W. Bruin J. (Amsterdam: Elsevier Science B.V), 571–582. 10.1016/S1572-4379(96)80036-4

[B40] Easterbrook M. A. (1996). “Damage and control of eriophyoid mites in apple and pear,” in *Eriophyoid Mites-Their Biology, Natural Enemies and Control*, eds Lindquist E. E. Sabelis M. W. Bruin J. (Amsterdam: Elsevier Science B.V), 527–541. 10.1016/S1572-4379(96)80033-9

[B41] Epstein A. H. Hill J. H. (1999). Status of rose rosette disease as a biological control for multiflora rose. *Plant Dis.* 83 92–101. 10.1094/PDIS.1999.83.2.92 30849819

[B42] Gamliel-Atinsky E. Freeman S. Maymon M. Belausov E. Ochoa R. Bauchan G. (2010). The role of eriophyoids in fungal pathogen epidemiology, mere association or true interaction? *Exp. Appl. Acarol.* 51 191–204. 10.1007/s10493-009-9302-y 19774470

[B43] Gantner M. (2009). The role of tannins in the resistance of hazelnut cultivated in poland to the major pests. *Acta Horticult.* 845 471–478. 10.17660/ActaHortic.2009.845.73

[B44] Gantner M. Najda A. (2013). Essential oils from buds and leaves of two hazelnut (*Corylus* L.) cultivars with different resistance to filbert big bud mite (*Phytoptus avellanae* Nal.) and filbert aphid (*Myzocallis coryli* Goetze). *Arthropod Plant Interact.* 7 659–666. 10.1007/s11829-013-9281-0

[B45] Gascuel Q. Diretto G. Monforte A. J. Fortes A. M. Granell A. (2017). Use of natural diversity and biotechnology to increase the quality and nutritional content of tomato and grape. *Front. Plant Sci.* 8:652. 10.3389/fpls.2017.00652 28553296 PMC5427129

[B46] Gatehouse J. A. (2002). Plant resistance towards insect herbivores: a dynamic interaction. *New Phytol.* 156 145–169. 10.1046/j.1469-8137.2002.00519.x 33873279

[B47] Glas J. J. Alba J. M. Simoni S. Villarroel C. A. Stoops M. Schimmel B. C. J. (2014). Defense suppression benefits herbivores that have a monopoly on their feeding site but can backfire within natural communities. *BMC Biol.* 12:98. 10.1186/s12915-014-0098-9 25403155 PMC4258945

[B48] Haque E. Taniguchi H. Hassan M. M. Bhowmik P. Karim M. R. Śmiech M. (2018). Application of CRISPR/Cas9 genome editing technology for the improvement of crops cultivated in tropical climates: recent progress, prospects, and challenges. *Front. Plant Sci.* 9:617. 10.3389/fpls.2018.00617 29868073 PMC5952327

[B49] Harvey T. L. Martin T. J. Seifers D. L. (2003). Resistance to the wheat curl mite (Acari: Eriophyidae) prevents loss in wheat yield. *J. Agric. Urban Entomol.* 20 7–10.

[B50] Harvey T. L. Seifers D. L. Martin T. J. Michaud J. P. (2005). Effect of resistance to wheat streak mosaic virus on transmission efficiency of wheat curl mites. *J. Agric. Urban Entomol.* 22 1–6.

[B51] Hassan M. Di Bello P. Keller K. E. Martin R. R. Sabanadzoivc S. Tzanetakis J. (2017). A new, widespread emaravirus discovered in blackberry. *Virus Res.* 235 1–5. 10.1016/j.virusres.2017.04.006 28396285

[B52] Hein G. L. French R. Siriwetwiwat B. Amrine J. W. (2012). Genetic characterization of north american populations of the wheat curl mite and dry bulb mite. *J. Econ. Entomol.* 105 1801–1808. 10.1603/EC11428 23156180

[B53] Javadi Khederi S. de Lillo E. Khanjani M. Gholami M. (2014). Resistance of grapevine to the erineum strain of *Colomerus vitis* (Acari: Eriophyidae) in western iran and its correlation with plant features. *Exp. Appl. Acarol.* 63 15–35. 10.1007/s10493-014-9778-y 24519017

[B54] Javadi Khederi S. Khanjani M. Gholami M. Bruno G. L. (2018a). Study of defense-related gene expression in grapevine infested by *Colomerus vitis* (Acari: Eriophyidae). *Exp. Appl. Acarol.* 75 25–40. 10.1007/s10493-018-0255-x 29611071

[B55] Javadi Khederi S. Khanjani M. Gholami M. de Lillo E. (2018b). Impact of the erineum strain of *Colomerus vitis* (Acari: Eriophyidae) on the development of plants of grapevine cultivars of iran. *Exp. Appl. Acarol.* 74 347–363. 10.1007/s10493-018-0245-z 29572700

[B56] Javadi Khederi S. Khanjani M. Gholami M. de Lillo E. (2018c). Sources of resistance to the erineum strain of *Colomerus vitis* (Acari: Eriophyidae) in grapevine cultivars. *Syst. Appl. Acarol.* 23 405–425. 10.1007/s10493-014-9778-y 29572700

[B57] Javadi Khederi S. Khanjani M. Gholami M. Panzarino O. de Lillo E. (2018d). Influence of the erineum strain of *Colomerus vitis* (Acari: Eriophyidae) on grape (*Vitis vinifera*) defense mechanisms. *Exp. Appl. Acarol.* 75 1–24. 10.1007/s10493-018-0252-0 29611069

[B58] Jonckheere W. Dermauw W. Khalighi M. Pavlidi N. Reubens W. Baggerman G. (2018). A gene family coding for salivary proteins (SHOT) of the polyphagous spider mite *Tetranychus urticae* exhibits fast host-dependent transcriptional plasticity. *Mol. Plant-Microbe Interact.* 31 112–124. 10.1094/MPMI-06-17-0139-R 29094648

[B59] Jonckheere W. Dermauw W. Zhurov V. Wybouw N. Van Den Bulcke J. Villarroel C. A. (2016). The salivary protein repertoire of the polyphagous spider mite *Tetranychus urticae*: a quest for effectors. *Mol. Cell. Proteomics* 15 3594–3613. 10.1074/mcp.M116.058081 27703040 PMC5141274

[B60] Jones A. T. (2000). Black currant reversion disease the probable causal agent, eriophyid mite vectors, epidemiology and prospects for control. *Virus Res.* 71 71–84. 10.1016/S0168-1702(00)00189-1 11137163

[B61] Jones A. T. (2002). Important virus diseases of *Ribes*, their diagnosis, detection and control. *Acta Horticult.* 585 279–285. 10.17660/ActaHortic.2002.585.45

[B62] Jones A. T. Brennan R. M. McGavin W. J. Lemmetty A. (1998). Galling and reversion disease incidence in a range of blackcurrant genotypes, differing in resistance to the blackcurrant gall mite (*Cecidophyopsis ribis*) and blackcurrant reversion disease. *Ann. Appl. Biol.* 133 375–384. 10.1111/j.1744-7348.1998.tb05837.x

[B63] Jones A. T. Kumar P. L. Saxena K. B. Kulkarni N. K. Muniyappa V. Waliyar F. (2004). Sterility mosaic disease on the “green plague” of pigeonpea: advances in understanding the etiology, transmission and control of a major virus disease. *Plant Dis.* 88 436–445. 10.1094/PDIS.2004.88.5.436 30812645

[B64] Jones R. A. C. Coutts B. A. Mackie A. E. Dwyer G. I. (2005). Seed transmission of wheat streak mosaic virus shown unequivocally in wheat. *Plant Dis.* 89 1048–1050. 10.1094/PD-89-1048 30791271

[B65] Karkute S. G. Singh A. K. Gupta O. P. Singh P. M. Singh B. (2017). CRISPR/Cas9 mediated genome engineering for improvement of horticultural crops. *Front. Plant Sci* 8:1635. 10.3389/fpls.2017.01635 28970844 PMC5609112

[B66] Keep E. Knight V. H. Parker J. H. (1982). Progress in the integration of characters in gall mite resistant black currants. *J. Hort. Sci.* 57 189–196. 10.1080/00221589.1982.11515039

[B67] Kiedrowicz A. Rector B. Denizhan E. SzydłĆo W. Skoracka A. (2014). Infestation of grasses by eriophyoid mites (Acari: Eriophyoidea) in Turkey. *Int. J. Acarol.* 40 421–427. 10.1080/01647954.2014.941004

[B68] Kiedrowicz A. Rector B. G. Lommen S. Kuczynski L. Szydo W. Skoracka A. (2017). Population growth rate of dry bulb mite, *Aceria tulipae* (Acariformes: Eriophyidae), on agriculturally important plants and implications for its taxonomic status. *Exp. Appl. Acarol.* 73 1–10. 10.1007/s10493-017-0173-3 28856573 PMC5602028

[B69] Kielkiewicz M. Soika G. Olszewska-Kaczynska I. (2011). A comparative evaluation of the consequences of *Phytoptus tetratrichus* nalepa (Acari: Eriophyoidea) feeding on the content and tissue distribution of polyphenolic compounds in leaves of different linden taxa. *Acarologia* 51 237–250. 10.1051/acarologia/20112007

[B70] Kitamura T. Kawai A. (2006). Difference of susceptibility to damage from tomato russet mite, *Aculops lycopersici* (Massee) (Acari: Eriophidae), among varieties within and between species in genus *Lycopersicon*. *Jap. J. Appl. Entomol. Zool.* 50 57–61. 10.1303/jjaez.2006.57

[B71] Knight R. L. Keep E. Briggs J. B. Parker J. (1974). Transference of resistance to black currant gall mite *Cecidophyopsis ribis*, from gooseberry to black currant. *Ann. Appl. Biol.* 76 123–130. 10.1111/j.1744-7348.1974.tb01362.x

[B72] Kulkarni N. K. Kumar P. L. Muniyappa V. Jones A. T. Reddy D. V. R. (2002). Transmission of pigeonpea sterility mosaic virus by the eriophyid mite, *Aceria cajani* (Acari: Arthropoda). *Plant Dis.* 86 1297–1302. 10.1111/mpp.12238 30818431

[B73] Kumar P. L. Fenton B. Duncan G. H. Jones A. T. Sreenivasulu P. Reddy D. V. R. (2001). Assessment of variation in *Aceria cajani* using analysis of rDNA ITS regions and scanning electron microscopy: implications for the variability observed in host plant resistance to pigeonpea sterility mosaic disease. *Ann. Appl. Biol.* 139 61–73. 10.1111/j.1744-7348.2001.tb00131.x

[B74] Kumar P. L. Latha T. K. S. Kulkarni N. K. Raghavendra N. Saxena K. B. Waliyar F. (2005). Broad based resistance to pigeonpea sterility mosaic disease in wild relatives of pigeonpea (Cajanus: Phaseoleae). *Ann. Appl. Biol.* 146 371–379. 10.1111/j.1744-7348.2005.040091.x

[B75] Lanoiselet V. M. Hind-Lanoiselet T. L. Murray G. M. (2008). Studies on the seed transmission of wheat streak mosaic virus. australas. *Plant Path.* 37 584–588. 10.1071/AP08059

[B76] Latha T. K. S. Doraiswamy S. (2008). Detection of pigeonpea sterility mosaic virus, the causal agent of sterility mosaic disease of pigeonpea in viruliferous mite vector by DAS ELISA and DIBA. *Arch. Phytopath. Plant Prot.* 41 537–541. 10.1080/03235400600940855

[B77] Lee S. K. Im J. K. Jung J. K. Kim D.-H. Lee J.-H. (2015). Flower and leaf damage of grapevines caused by the grape rust mite, *Calepitrimerus vitis* (Nalepa). *J. Asia Pac. Ent.* 18 51–54. 10.1016/j.aspen.2014.12.001

[B78] Lee S. K. Im J. K. Lee H. Lee J.-H. (2018). Predicting early spring emergence and late season overwintering movement of *Calepitrimerus vitis* (Nalepa) (Acari: Eriophyidae) in grapevine. *J. Asia Pac. Ent.* 21 1182–1185. 10.1016/j.aspen.2018.08.015

[B79] Lemmetty A. Tikkanen M. Tuovinen T. Lehto K. (2004). Identification of different *Cecidophyopsis* mites on ribes in finland. *Acta Horticult.* 656 115–118. 10.17660/ActaHortic.2004.656.17 7663755

[B80] Li H. J. Conner R. L. Chen Q. Jia X. Li H. Graf R. J. (2002). Different reactions to the wheat curl mite and wheat streak mosaic virus in various wheat-*Haynaldia villosa* 6V and 6VS lines. *Plant Dis.* 86 423–428. 10.1094/PDIS.2002.86.4.423 30818719

[B81] Lindquist E. E. Sabelis M. W. Bruin J. (1996). *Eriophyoid Mites-Their Biology, Natural Enemies and Control. World Crop Pests, Volume* 6. Amsterdam: Elsevier Science Publishers.

[B82] Liu J. Legarrea S. Kant M. R. (2017). Tomato reproductive success is equally affected by herbivores that induce or that suppress defenses. *Front. Plant Sci.* 8:2128. 10.3389/fpls.2017.02128 29326739 PMC5733352

[B83] Lorenzon M. Pozzebon A. Duso C. (2012). Effects of potential food sources on biological and demographic parameters of the predatory mites *Kampimodromus aberrans*, *Typhlodromus pyri* and *Amblyseius andersoni*. *Exp. Appl. Acarol.* 58 259–278. 10.1007/s10493-012-9580-7 22836719

[B84] Malagnini V. de Lillo E. Saldarelli P. Beber R. Duso C. Raiola A. (2016). Transmission of grapevine pinot gris virus by colomerus vitis (Acari: Eriophyidae) to grapevine. *Arch. Virol.* 161 2595–2599. 10.1007/s00705-016-2935-3 27344161

[B85] Malik R. Brown-Guedira G. L. Smith C. M. Harvey T. L. Gill B. S. (2003). Assessment of *Aegilops tauschii* for resistance to biotypes of wheat curl mite (Acari: Eriophyidae). *J. Econ. Entomol.* 96 1329–1333. 10.1093/jee/96.4.1329 14503608

[B86] Maoz Y. Gal S. Argov Y. Domeratzky S. Coll M. Palevsky E. (2016). Intraguild interactions among specialised pollen feeders and generalist phytoseiids and their effect on citrus rust mite suppression. *Pest Man Sci.* 72 940–949. 10.1002/ps.4073 26130195

[B87] Martel C. Zhurov V. Navarro M. Martinez M. Cazaux M. Auger P. (2015). Tomato whole genome transcriptional response to *Tetranychus urticae* identifies divergence of spider mite-induced responses between tomato and *Arabidopsis*. *Mol. Plant Microbe. Interact.* 28 343–361. 10.1094/MPMI-09-14-0291-FI 25679539

[B88] Martin T. J. Harvey T. L. Bender C. G. Seifers D. L. Hatchett J. H. (1983). Wheat curl mite resistance wheat germplasm. *Crop. Sci.* 23:89 10.2135/cropsci1983.0011183X002300040075x

[B89] Maurya R. K. Kumar K. Kumar R. Singh M. (2017). Transmission of pigeon pea sterility mosaic virus and management of sterility mosaic disease of pigeonpea by different acaricides under middle IGP of bihar. *Int. J. Curr. Microbiol. App. Sci.* 6 3711–3716. 10.20546/ijcmas.2017.608.448

[B90] Mayer A. M. (2006). Polyphenol oxidases in plants and fungi: going places? A review. *Phytochemistry* 67 2318–2331. 10.1016/j.phytochem.2006.08.006 16973188

[B91] Mazeikiene I. Bendokas V. Baniulis D. Staniene G. Juskyte D. A. Sasnauskas A. (2017). Genetic background of resistance to gall mite in *Ribes* species. *Agric. Food Sci.* 26 111–117. 10.23986/afsci.59410

[B92] Mazeikiene I. Bendokas V. Stanys V. Siksnianas T. (2012). Molecular markers linked to resistance to the gall mite in blackcurrant. *Plant Breed.* 131 762–766. 10.1007/s00122-008-0889-x 18813905

[B93] McMechan A. J. (2012). *Transmission of Triticum Mosaic Virus and its Impact on the Biology of the Wheat Curl Mite Aceria Tosichella Keifer (Eriophyidae), and an Evaluation of Management Tactics for the Wheat Curl Mite and the Wheat-mite-Virus Complex.* Ph.D, thesis, University of Nebraska, Lincoln.

[B94] McMechan A. J. Tatineni S. French R. Hein G. L. (2014). Differential Transmission of *Triticum* mosaic virus by wheat curl mite populations collected in the great plains. *Plant Dis.* 98 806–810. 10.1094/PDIS-06-13-0582-RE 30708632

[B95] Miller A. D. Skoracka A. Navia D. Mendonca R. S. de SzydłĆo W. Schultz M. B. (2013). Phylogenetic analyses reveal extensive cryptic speciation and host specialization in an economically important mite taxon. *Mol. Phylogenet. Evol.* 66 928–940. 10.1016/j.ympev.2012.11.021 23246929

[B96] Mitchell C. Brennan R. M. Cross J. V. Johnson S. N. (2011). Arthropod pests of currant and gooseberry crops in the U.K.: their biology, management and future prospects. *Agric. For. Entomol.* 13 221–237. 10.1111/j.1461-9563.2010.00513.x

[B97] Mitchell C. Brennan R. M. Graham J. Karley A. J. (2016). Plant defense against herbivorous pests: exploiting resistance and tolerance traits for sustainable crop protection. *Front. Plant Sci.* 7:1132. 10.3389/fpls.2016.01132 27524994 PMC4965446

[B98] Monfreda R. de Lillo E. (2010). Attuali conoscenze sulle secrezioni salivari negli Acari Eriophyoidea. Atti Accademia Nationally di Entomologia. *Rendiconti* 53 379–388.

[B99] Monfreda R. Spagnuolo M. (2004). Enzyme activity of an eriophyoid ‘salivary’ secretion: preliminary report of polygalacturonase. *Phytophaga* 14 611–614. 22230313

[B100] Murugan M. Cardona P. S. Duraimurugan P. Whitfield A. E. Schneweis D. Starkey S. (2011). Wheat curl mite resistance: interactions of mite feeding with wheat streak mosaic virus infection. *J. Econ. Entomol.* 104 1406–1414. 10.1603/EC11112 21882710

[B101] Navia D. de Mendonça R. S. Skoracka A. SzydłĆo W. Knihinicki D. Hein G. L. (2013a). Wheat curl mite, *Aceria tosichella*, and transmitted viruses: an expanding pest complex affecting cereal crops. *Exp. Appl. Acarol.* 59 95–143. 10.1007/s10493-012-9633-y 23179064

[B102] Navia D. Gondim M. G. C. Aratchige N. S. de Moraes G. J. (2013b). A review of the status of the coconut mite, *Aceria guerreronis* (Acari: Eriophyidae), a major tropical mite pest. *Exp. Appl. Acarol.* 59 67–94. 10.1007/s10493-012-9634-x 23192330

[B103] Navia D. Ochoa R. Welbourn C. Ferragut F. (2010). Adventive eriophyoid mites: a global review of their impact, pathways, prevention and challenges. *Exp. Appl. Acarol.* 51 225–255. 10.1007/978-90-481-9562-6_12 19844795

[B104] Oldfield G. N. Proeseler G. (1996). “Eriophyoid mites as vectors of plant pathogens,” in *Eriophyoid Mites-Their Biology, Natural Enemies and Control*, eds Lindquist E. E. Sabelis M. W. Bruin J. (Amsterdam: Elsevier Science B.V), 259–273. 10.1016/S1572-4379(96)80017-0

[B105] Özman S. K. Toros S. (1996). “Damage caused by *Phytoptus avellanae* Nal. and *Cecidophyopsis vermiformis* Nal.(Eriophyoidea: Acarina) in hazelnut,” in *Proceeding of IV International Symposium on Hazelnut* Vol. 445 (Anakara), 537–544.

[B106] Pallavi M. S. Ramappa H. K. (2014). Population dynamics of pigeonpea sterility mosaic virus disease vector *Aceria cajani*. *Mysore J. Agric. Sci.* 48 394–399.

[B107] Paponova S. S. Chetverikov P. E. Pautov A. A. Yakovleva O. V. Zukoff S. N. Vishnyakov A. E. (2018). Gall mite *Fragariocoptes setiger* (Eriophyoidea) changes leaf developmental program and regulates gene expression in the leaf tissues of *Fragaria viridis* (Rosaceae). *Ann. Appl. Biol.* 172 33–46. 10.1111/aab.12399

[B108] Patankar R. Thomas S. C. Smith S. M. (2011). A gall-inducing arthropod drives declines in canopy tree photosynthesis. *Oecologia* 167 701–709. 10.1007/s00442-011-2019-8 21618011

[B109] Petanović R. Chuang W. P. Rojas L. M. A. Khalaf L. K. Zhang G. R. Fritz A. K. (2017). Wheat genotypes with combined resistance to wheat curl mite, wheat streak mosaic virus, wheat mosaic virus, and triticum mosaic virus. *J. Econ. Entomol.* 110 711–718. 10.1093/jee/tow255 28087646

[B110] Petanović R. Kielkiewicz M. (2010a). Plant-eriophyoid mite interactions: cellular biochemistry and metabolic responses induced in mite-injured plants. *Exp. Appl. Acarol.* 51 61–80. 10.1007/s10493-010-9351-2 20229098

[B111] Petanović R. Kielkiewicz M. (2010b). Plant-eriophyoid mite interactions: specific and unspecific morphological alterations. Part II. *Exp. Appl. Acarol.* 51 81–91. 10.1007/978-90-481-9562-6_5 20012342

[B112] Peterson R. K. D. Varella A. C. Higley L. G. (2017). Tolerance: the forgotten child of plant resistance. *PeerJ* 5:e3934. 10.7717/peerj.3934 29062607 PMC5647859

[B113] Petkau A. Chelack W. S. Pleskach S. D. Meeker B. E. Brady C. M. (1975). Radioprotection of mice by superoxide dismutase. *Biochem. Biophys. Res. Commun.* 65 886–893. 10.1016/S0006-291X(75)80468-2 1156422

[B114] Ranabhat N. B. Seipel T. Lehnhoff E. A. (2018). Temperature and alternative hosts influence *Aceria tosichella* infestation and wheat streak mosaic virus infection. *Plant Dis.* 102 546–551. 10.1094/PDIS-06-17-0782-RE 30673491

[B115] Reddy M. V. Nene Y. L. (1980). Influence of sterility mosaic virus resistant pigeon peas on multiplication of the mite vector. *Indian Phytopathol.* 33 61–63.

[B116] Richardson K. Miller A. D. Hoffmann A. A. Larkin P. (2014). Potential new sources of wheat curl mite resistance in wheat to prevent the spread of yield-reducing pathogens. *Exp. Appl. Acarol.* 64 1–19. 10.1007/s10493-014-9808-9 24705793

[B117] Rioja C. Zhurov V. Bruinsma K. Grbic M. Grbic V. (2017). Plant-herbivore interactions: a case of an extreme generalist, the two-spotted spider mite *Tetranychus urticae*. *Mol. Plant Microbe Interact.* 30 935–945. 10.1094/MPMI-07-17-0168-CR 28857675

[B118] Royalty R. N. Perring T. M. (1996). “Nature of damage and its assessment,” in *Eriophyoid Mites-Their Biology, Natural Enemies and Control*, eds Lindquist E. E. Sabelis M. W. Bruin J. (Amsterdam: Elsevier Science B.V), 493–512. 10.1016/S1572-4379(96)80031-5

[B119] Royalty R. N. Perring T. M. (1988). Morphological analysis of damage to tomato leaflets by tomato russet mite (Acari: Eriophyidae). *J. Econ. Entomol.* 81 816–820.

[B120] Ryan C. A. (2000). The systemin signalling pathway: differential activation of plant defensive genes. *Bioch. Bioph. Acta* 1477 112–121. 10708853 10.1016/s0167-4838(99)00269-1

[B121] Salinas M. Capel C. Alba J. M. Mora B. Cuartero J. Fernández-Muñoz R. (2013). Genetic mapping of two QTL from the wild tomato *Solanum pimpinellifolium* L. controlling resistance against two-spotted spider mite *(Tetranychus urticae Koch)*. *Theor. Appl. Genet.* 126 83–92. 10.1007/s00122-012-1961-0 22903693

[B122] Santamaria M. E. Diaz I. Martinez M. (2018). Dehydration stress contributes to the enhancement of plant defense response and mite performance on barley. *Front. Plant Sci.* 9:458. 10.3389/fpls.2018.00458 29681917 PMC5898276

[B123] Santamaria M. E. Martinez M. Arnaiz A. Ortego F. Grbic V. Diaz I. (2017). MATI, a novel protein involved in the regulation of herbivore-associated signaling pathways. *Front. Plant Sci.* 8:975. 10.3389/fpls.2017.00975 28649257 PMC5466143

[B124] Santamaria M. E. Martínez M. Cambra I. Grbic V. Diaz I. (2013). Understanding plant defence responses against herbivore attacks: an essential first step towards the development of sustainable resistance against pests. *Transgenic Res.* 22 697–708. 10.1007/s11248-013-9725-4 23793555

[B125] Sapakova E. Svobodova Z. Sefrova H. Hasikova L. (2015). “Infestation by *Aceria tulipae* (Keifer) (Acari: Eriophyidae), Economy and Marketing of Growing Garlic in Regional Agricultural Areas,” in *Proceedings of the IX International. Conference on Appiled Business Research*, (Brno).

[B126] Sasnauskas A. Trajkovski V. Strautina S. Tikhonova O. Siksniasas T. Rubinskiene M. (2009). Evaluation of blackcurrant cultivars and perspective hybrids in Lith. *Agronom. Res.* 7 737–743.

[B127] Schiffer M. Umina P. Carew M. Hoffmann A. Rodoni B. Miller A. (2009). The distribution of wheat curl mite (*Aceria tosichella*) lineages in Australia and their potential to transmit wheat streak mosaic virus. *Ann. Appl. Biol.* 155 371–379. 10.1111/j.1744-7348.2009.00349.x

[B128] Shalini K. V. Manjunatha S. Lebrun P. Berger A. Baudouin L. Pirany N. (2007). Identification of molecular markers associated with mite resistance in coconut (*Cocos nucifera* L.). *Genome* 50 35–42. 10.1139/g06-136 17546069

[B129] Shi A. Tomczyk A. (2001). Impact of feeding of eriophyid mite *Epitrimerus gibbosus* (Nalepa) (Acari: Eriophyoidea) on some biochemical components of blackberry (*Rubus* spp.). *Bull. Polish Acad. Sci. Biol. Sci.* 49 41–47.

[B130] Simoni S. Angeli G. Baldessari M. Duso C. (2018). Effects of *Aculus schlechtendali* (Acari: Eriophyidae) population densities on golden delicious apple production. *Acarologia* 58 133–144.

[B131] Siriwetwiwat B. (2006). *Interactions Between the Wheat Curl Mite, Aceria Tosichella Keifer (Eriophyidae), and Wheat Streak Mosaic Virus and Distribution of Wheat Curl Mite Biotypes in the Field.* Ph.D. thesis, University of Nebraska, Lincoln.

[B132] Skoracka A. (2009). Description of *Abacarus lolii* n. sp. *(Acari: Prostigmata: Eriophyoidea*), a cryptic species within a grass-feeding Abacarus complex. *Int. J. Acarol.* 35 405–417. 10.1080/01647950903292764

[B133] Skoracka A. Dabert M. (2010). The cereal rust mite *Abacarus hystrix* (Acari: Eriophyoidea) is a complex of species: evidence from mitochondrial and nuclear DNA sequences. *Bull. Entomol. Res.* 100 263–272. 10.1017/S0007485309990216 19671206

[B134] Skoracka A. Kuczyński L. (2006). Is the cereal rust mite, *Abacarus hystrix* really a generalist? - testing, colonization performance on novel hosts. *Exp. Appl. Acarol.* 38 1–13. 10.1007/s10493-005-6077-7 16550330

[B135] Skoracka A. Kuczynski L. de Mendonca Santos R. Dabert M. Szydlo W. Knihinicki D. (2012). Cryptic species within the wheat curl mite *Aceria tosichella* (Keifer) (Acari : Eriophyoidea), revealed by mitochondrial, nuclear and morphometric data. *Inv. Sys.* 26 417–433. 10.1071/IS11037

[B136] Skoracka A. Kuczyński L. Rector B. Amrine J. W. Jr. (2014a). Wheat curl mite and dry bulb mite: untangling a taxonomic conundrum through a multidisciplinary approach. *Biol. J. Linn. Soc.* 111 421–436. 10.1111/bij.12213

[B137] Skoracka A. Rector B. Kuczynski L. Szydlo W. Hein G. French R. (2014b). Global spread of wheat curl mite by its most polyphagous and pestiferous lineages. *Ann. Appl. Biol.* 165 222–235. 10.1111/aab.12130

[B138] Skoracka A. Kuczyński L. Szydlo W. Rector B. (2013). The wheat curl mite *Aceria tosichella* (Acari: Eriophyoidea) is a complex of cryptic lineages with divergent host ranges: evidence from molecular and plant bioassay data. *Biol. J. Linn. Soc.* 109 165–180. 10.1111/bij.12024

[B139] Skoracka A. Lewandowski M. Rector B. G. Szydlo W. Kuczyński L. (2017). Spatial and host-related variation in prevalence and population density of wheat curl mite (*Aceria tosichella*) cryptic genotypes in agricultural landscapes. *PLoS One* 12:e0169874. 10.1371/journal.pone.0169874 28099506 PMC5242520

[B140] Skoracka A. Magalhães S. Rector B. G. Kuczyński L. (2015). Cryptic speciation in the acari: a function of species lifestyles or our ability to separate species? *Exp. Appl. Acarol.* 67 165–182. 10.1007/s10493-015-9954-8 26209969 PMC4559570

[B141] Skoracka A. Rector B. G. Hein G. L. (2018). The interface between wheat and the wheat curl mite, *Aceria tosichella*, the primary vector of globally important viral diseases. *Front. Plant Sci.* 9:1098. 10.3389/fpls.2018.01098 30100916 PMC6072864

[B142] Skoracka A. Smith L. Oldfield G. Cristofaro M. Amrine J. W. Jr. (2010). Host-plant specificity and specialization in eriophyoid mites and their importance for the use of eriophyoid mites as biocontrol agents of weeds. *Exp. Appl. Acarol.* 51 93–113. 10.1007/s10493-009-9323-6 19789985

[B143] Smith L. de Lillo E. Amrine J. W. Jr. (2010). Effectiveness of eriophyid mites for biological control of weedy plants and challenges for future research. *Exp. Appl. Acarol.* 51 115–149. 10.1007/s10493-009-9299-2 19760101

[B144] Soika G. Tomczyk A. Kozak M. (2017). Biochemical reaction of tilia leaves on infestation by some species of gall-inducing eriophyoid mites. *Int. J. Acarol.* 43 16–21. 10.1080/01647954.2016.1223168

[B145] Sousa A. S. G. Argolo P. A. Gondim M. G. C. de Moraes G. J. Oliveira A. R. (2017). Influence of fruit age of the brazilian green dwarf coconut on the relationship between *Aceria guerreronis* population density and percentage of fruit damage. *Exp. Appl. Acarol.* 72 329–337. 10.1007/s10493-017-0152-8 28831715

[B146] Sperotto R. A. Buffon G. Schwambach J. Ricachenevsky F. K. (2018). Crops responses to mite infestation: it ’ s time to look at plant tolerance to meet the farmers’ needs. *Front. Plant Sci.* 9:556. 10.3389/fpls.2018.00556 29740472 PMC5928466

[B147] Stalažs A. Moroèko-Bičevska I. (2016). Species identification, host range and diversity of *Cecidophyopsis* mites (Acari: Trombidiformes) infesting *Ribes* in latvia. *Exp. Appl. Acarol.* 69 129–153. 10.1007/s10493-016-0024-7 26914359

[B148] Stenberg J. A. Muola A. (2017). How should plant resistance to herbivores be measured? *Front. Plant Sci.* 8:663. 10.3389/fpls.2017.00663 28491077 PMC5405324

[B149] Stoeckli S. Mody K. Dorn S. Kellerhals M. (2011). Association between herbivore resistance and fruit quality in apple. *HortScience* 46 12–15.

[B150] Stoeckli S. Mody K. Patocchi A. Kellerhals M. Dorn S. (2009). Rust mite resistance in apple assessed by quantitative trait loci analysis. *Tree Gen. Genom* 5 257–267. 10.1007/s11295-008-0186-5

[B151] Storey K. B. (1996). Oxidative stress: animal adaptations in nature. *Braz. J. Med. Biol. Res.* 29 1715–1733. 9222437

[B152] Stout M. J. Workman K. V. Duffey S. S. (1996). Identity, spatial distribution, and variability of induced chemical responses in tomato plants. *Entomol. Exp. Appl.* 79 255–271. 10.1111/j.1570-7458.1996.tb00834.x

[B153] Suzuki T. Nunes M. A. España M. U. Namin H. H. Jin P. Bensoussan N. (2017). RNAi-based reverse genetics in the chelicerate model *Tetranychus urticae*: a comparative analysis of five methods for gene silencing. *PLoS One* 12:e0180654. 10.1371/journal.pone.0180654 28704448 PMC5507529

[B154] Takayama K. Iyoki S. Takahashi N. Nishina H. Van Henten E. J. (2013). Plant diagnosis by monitoring plant smell: detection of russet mite damages on tomato plants. *IFAC Proc. Vol.* 46 68–70. 10.3182/20130327-3-JP-3017.00018

[B155] Tandon P. (1985). Peroxidase-catalyzed IAA-oxidation in presence of cofactors and auxin protectors isolated from *Eriophyes* incited *Zizyphus* gall tissue. *Cecid. Int.* 6 69–81.

[B156] Tandon P. Arya H. C. (1980). Presence of auxin protectors in *Eriophyes* induced *Zizyphus* stem galls. *Experientia* 36 958–959. 10.1007/BF01953817

[B157] Thipyapong P. Steffens J. C. (1997). Differential response of the polyphenol oxidase F promoter to injuries and wound signals. *Plant Physiol.* 115 409–418. 10.1104/pp.115.2.409 12223816 PMC158498

[B158] Ueckermann E. A. (2010). Special issue: eriophyoid mites: progress and prognoses – Preface. *Exp. Appl. Acarol.* 51 1–2. 10.1007/s10493-010-9345-0 20186463

[B159] van Houten Y. M. Glas J. J. Hoogerbrugge H. Rothe J. Bolckmans K. J. F. Simoni S. (2013). Herbivory-associated degradation of tomato trichomes and its impact on biological control of *Aculops lycopersici*. *Exp. Appl. Acarol.* 60 127–138. 10.1007/s10493-012-9638-6 23238958 PMC3641295

[B160] Van Leeuwen T. Dermauw W. (2016). The molecular evolution of xenobiotic metabolism and resistance in chelicerate mites. *Annu. Rev. Entomol.* 61 475–498. 10.1146/annurev-ento-010715-023907 26982444

[B161] Van Leeuwen T. Witters J. Nauen R. Duso C. Tirry L. (2010). The control of eriophyoid mites: state of the art and future challenges. *Exp. Appl. Acarol.* 51 205–224. 10.1007/s10493-009-9312-9 19768561

[B162] Villarroel C. A. Jonckheere W. Alba J. M. Glas J. J. Dermauw W. Haring M. A. (2016). Salivary proteins of spider mites suppress defenses in *Nicotiana benthamiana* and promote mite reproduction. *Plant J.* 86 119–131. 10.1111/tpj.13152 26946468

[B163] Walling L. L. (2000). The myriad plant responses to herbivores. *J. Plant Growth Regul.* 19 195–216. 11038228 10.1007/s003440000026

[B164] Walton V. M. Dreves A. J. Coop A. B. Jones G. V. Skinkis P. A. (2010). Developmental parameters and seasonal phenology of *Calepitrimerus vitis* (Acari: Eriophyidae) in wine grapes of western oregon. *Environ. Entomol.* 39 2006–2016. 10.1603/EN09197 22182568

[B165] Walton V. M. Dreves A. J. Gent D. H. James D. G. Martin R. R. Chambers U. (2007). Relationship between rust mites, *Calepitrimerus vitis* (Nalepa), bud mites *Colomerus vitis* (Pachenstecher) (Acari: Eriophyidae), and short shoot syndrome in oregon vineyards. *Int. J. Acarol.* 33 307–318. 10.1080/01647950708683691

[B166] Westphal E. Manson D. C. M. (1996). “Feeding effects on host plants: gall formation and other distortions,” in *Eriophyoid Mites-Their Biology, Natural Enemies and Control*, eds Lindquist E. E. Sabelis M. W. Bruin J. (Amsterdam: Elsevier Science B.V), 231–242. 10.1016/S1572-4379(96)80014-5

[B167] Wosula E. N. McMechan A. J. Hein G. L. (2015). The effect of temperature, relative humidity, and virus infection status on off-host survival of the wheat curl mite (Acari: Eriophyidae). *J. Econ. Entomol.* 108 1545–1552. 10.1093/jee/tov185 26470294

[B168] Ximenez-Embun M. G. Glass J. J. Ortego F. Alba J. M. Castanera P. Kant M. R. (2017). Drought stress promotes the colonization success of a herbivorous mite that manipulates plant defenses. *Exp. Appl. Acarol.* 73 297–315. 10.1007/s10493-017-0200-4 29188401 PMC5727147

[B169] Zanolli P. Fabris B. Pozzebon A. Duso C. (2010). Effects of natural derived products on the tomato russet mite aculops lycopersici. in *Proceedings of the IPM Innovation in Europe*, Poznan.

